# Microbioreactor Arrays for Full Factorial Screening of Exogenous and Paracrine Factors in Human Embryonic Stem Cell Differentiation

**DOI:** 10.1371/journal.pone.0052405

**Published:** 2012-12-26

**Authors:** Drew M. Titmarsh, James E. Hudson, Alejandro Hidalgo, Andrew G. Elefanty, Edouard G. Stanley, Ernst J. Wolvetang, Justin J. Cooper-White

**Affiliations:** 1 Australian Institute for Bioengineering & Nanotechnology, The University of Queensland, St. Lucia, Queensland, Australia; 2 Murdoch Childrens Research Institute, The Royal Children’s Hospital, Flemington Road, Parkville, Victoria, Australia; 3 Monash Immunology and Stem Cell Laboratories, Monash University, Clayton, Victoria, Australia; 4 School of Chemical Engineering, The University of Queensland, St. Lucia, Queensland, Australia; University of Cincinnati, United States of America

## Abstract

Timed exposure of pluripotent stem cell cultures to exogenous molecules is widely used to drive differentiation towards desired cell lineages. However, screening differentiation conditions in conventional static cultures can become impractical in large parameter spaces, and is intrinsically limited by poor spatiotemporal control of the microenvironment that also makes it impossible to determine whether exogenous factors act directly or through paracrine-dependent mechanisms. We detail here the development of a continuous flow microbioreactor array platform that combines full-factorial multiplexing of input factors with progressive accumulation of paracrine factors through serially-connected culture chambers, and further, the use of this system to explore the combinatorial parameter space of both exogenous and paracrine factors involved in human embryonic stem cell (hESC) differentiation to a MIXL1-GFP^+^ primitive streak-like population. We show that well known inducers of primitive streak (BMP, Activin and Wnt signals) do not simply act directly on hESC to induce MIXL1 expression, but that this requires accumulation of surplus, endogenous factors; and, that conditioned medium or FGF-2 supplementation is able to offset this. Our approach further reveals the presence of a paracrine, negative feedback loop to the MIXL1-GFP^+^ population, which can be overcome with GSK-3β inhibitors (BIO or CHIR99021), implicating secreted Wnt inhibitory signals such as DKKs and sFRPs as candidate effectors. Importantly, modulating paracrine effects identified in microbioreactor arrays by supplementing FGF-2 and CHIR in conventional static culture vessels resulted in improved differentiation outcomes. We therefore demonstrate that this microbioreactor array platform uniquely enables the identification and decoding of complex soluble factor signalling hierarchies, and that this not only challenges prevailing strategies for extrinsic control of hESC differentiation, but also is translatable to conventional culture systems.

## Introduction

Human pluripotent stem cells (hPSCs) [1], [2] and their differentiated progeny are attractive cell sources for application in regenerative medicine, as tools for pre-clinical drug screening, and as disease and developmental models. Realising the promise of these cells in these applications is predicated on the ability to effectively direct both their undifferentiated expansion and differentiation into desired cell lineages, in order to generate large numbers of well-defined cell populations. Throughout early development, a complex interplay of multiple microenvironmental stimuli and individual cell states control stem cell fate decisions that lead to morphogenetic patterning of heterogeneous, organised tissues. The deconstruction of these complex developmental processes, and application of the key drivers in efficiently and robustly directing hPSC differentiation into appreciable numbers of a target cell type, require precise control over microenvironmental parameters [3]. Identification of the relevant extrinsic factors, timing of treatment, effective concentrations and best-performing combinations of these variables can quickly become an impractical exercise, not only because of the throughput and cost limitations of screening factors in conventional static culture systems, but also because such systems impose considerable spatiotemporal fluctuations in levels of nutrients and metabolic waste products, supplemented exogenous factors, and undefined endogenous factors produced by a dynamic continuum of cell types in the culture, making them generally unsuitable for accurately probing how relative levels of multiple microenvironmental stimuli direct stem cell fate.

Currently, hPSC differentiation protocols rely on formation of embryoid bodies (EBs) or differentiation in static 2D cultures. Embryoid body (EB)-based differentiation protocols have shown success in generating differentiated cell types of interest, however they are heterogeneous structures [4] containing mixtures of cell types, and have differentiation outcomes highly dependent on aggregate size [5], which implicates microenvironmental parameters such as local cell density and endogenous factor accumulation [6] as being critical determinants. For these reasons the emergence of a target phenotype may depend on the presence of a secondary phenotype supporting its development, necessitating strategies to isolate target cells. While forced aggregation of wildtype stimulator and reporter gene-marked responder mouse embryonic stem cells (mESCs) was able to dissect endogenous signals with some success [7], such an approach still masks internal spatiotemporal variations in microenvironmental composition, making it difficult to directly link defined stimuli to specific differentiation outcomes.

The primitive streak (PS) is a pertinent example of a spatio-temporally transient, *in vivo* structure arising during gastrulation, which is marked by the transcription factor MIXL1 and contains mesendodermal elements which eventually give rise to, for example, cardiac, renal and haematopoietic lineages. Efficient and homogeneous production of defined cell populations from such lineages requires exquisite control over differentiation outcomes across a number of distinct developmental stages. Since embryonic development has so far provided the best indication of the signals and intermediate cell types which are required for *in vitro* differentiation, and protocols based on developmental processes have generally met with the most success [3], generation of mature or progenitor cells of cardiac and haematopoietic lineages from hPSCs is thought to require passage through a transient PS-like stage, or a specific subpopulation of PS which is patterned towards a specific lineage outcome in part by diffusible, paracrine signals. Thus, there is a clear need for an experimental platform that allows investigation of the involvement of paracrine factors in differentiation processes, and provides separation, control and/or visualisation of these effects with reasonable throughput. Very recently, a number of microfluidic cell culture systems, utilising programmed medium exchange or continuous medium flow, have provided insight into the impacts of paracrine effects on culture outcomes of mouse PSCs [8], [9], [10], [11]. Such microtechnologies are providing parallelised screening platforms for microenvironmental conditions [12], and due to the explicit control of the microenvironmental parameter space that these devices afford, the data they produce can be paired with mathematical and computational models that allow deconvolution of paracrine processes [8], [10].

In this present work, by leveraging the advantageous features of microscale platforms, we have designed, fabricated and validated a scalable, continuous flow, full-factorial microbioreactor array platform to deconstruct the complexity of microenvironmental control of stem cell fate (Methods). The platform utilises continuous medium perfusion to allow provision of exogenous factors under “blank slate”, spatiotemporally-controlled microenvironmental conditions, with subsequent graded introduction of paracrine effects, allowing simultaneous evaluation of combinations of both types of stimuli, while dynamic reporter gene expression and/or *in situ* immunostaining at the experiment endpoint provide real-time and/or end point readouts, respectively, of cell phenotype. Since the formation and specification of the PS is thought to be determined for a large part by diffusible BMP, Activin and Wnt signals, PS definition was an ideal process for testing the utility of the microbioreactor array platform in applying various combinations of exogenous factors under steady state conditions and simultaneously screening for paracrine signalling-dependent outcomes. Using this platform, we demonstrate direct visualisation of paracrine-dependent phenotype outcomes, as well as modulation of paracrine signals involved in both induction and negative feedback regulation of the formation of a PS-like population. Using this platform to screen paracrine-dependent outcomes under various exogenous factor treatment regimes, we thereafter provide clues to the identity of the molecules involved, and confirm that insights gained through optimisation of paracrine-depenedent outcomes is translatable to improve differentiation outcomes in standard culture formats.

## Results

### Development and Validation of a Highly Integrated and Scalable, Full Factorial Microbioreactor Array Platform for hPSCs

**Figure 1:**
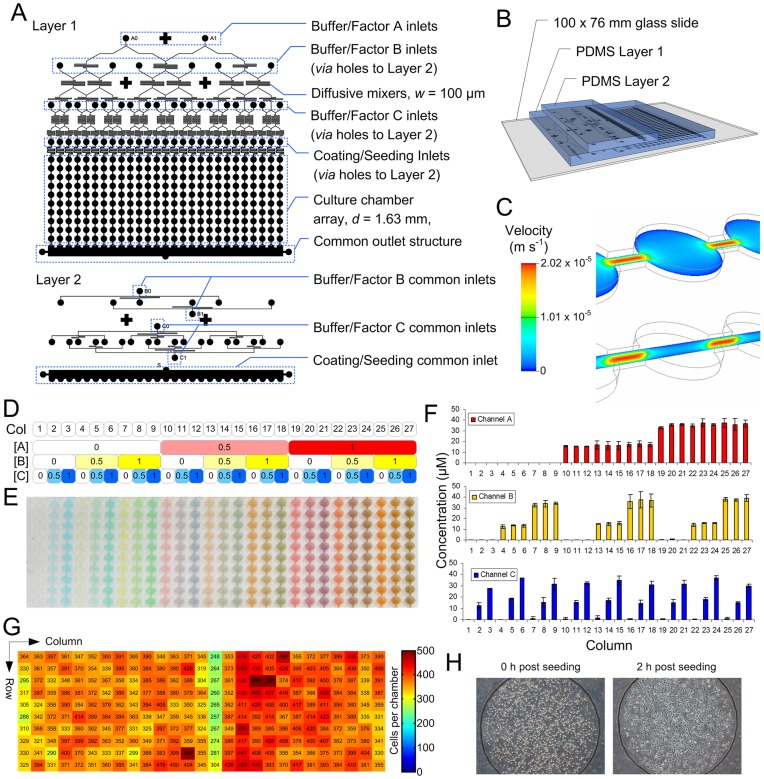
Microbioreactor array design and validation. **A** Microbioreactor array photomask design, with key features marked. **B** Perspective schematic view showing assembly of glass substrate and PDMS structural layers 1 and 2. *Via* holes join microchannel structures between layers. **C** Computational fluid dynamic model of velocity field through centreline planes at nominal operating conditions. Chambers are 1.63 mm in diameter and 250 µm high. **D** Design normalised concentrations of factors in each column, corresponding to panels E and F. Stock factor and buffer solutions are provided at normalised concentrations of 3 and 0, respectively, to allow for subsequent dilution. **E** Photograph of microbioreactor array filled with red, yellow and blue food dyes (representing factors A1, B1 & C1, respectively), and mixed with PBS (buffers A0, B0, & C0). **F** Fluorimetric quantification of soluble factor levels in each column. Stock solution of 40 kDa FITC-dextran was provided at 100 µM, therefore the design concentration levels are 0, 16.7 and 33.3 µM. Bars represent mean ± SD of 2 independently fabricated devices. **G** Heatmap of number of nuclei in each chamber, with individual numbers marked. Cell numbers do not represent densities used in microbioreactor experiments. **H** Phase contrast images of hESCs imaged 0 and 2 h after seeding into microbioreactor arrays. Chambers are ∼1.63 mm in diameter.

The microbioreactor array generates all combinations of 3 concentrations each of 3 soluble factors (a full factorial array; 3^3^ = 27 distinct conditions in total), from only 6 fluidic inputs. Our resistive flow design for the array simultaneously allowed aliquoting, mixing, and full factorial multiplexing of exogenous factors to be encoded solely by the geometry of the microfluidic channel network (Figure 1A,B,D), eliminating the need for integrated valves and thereby greatly reducing the amount of peripheral equipment required for operation while still achieving complex flow control outcomes. Importantly, this flexible architecture is readily scalable to *a* parallel and/or serial replicate chambers, *b* concentration levels, and *c* exogenous factors, giving a total of *ab^c^* experimental points while requiring only 2*c* (or minimally *c*+1 if all buffers are the same) external connections, and 2 physical device layers [13], presenting an important scalability advantage over valved microfluidic systems. After multiplexing, the array supplies exogenous factors to a grid of 270 culture chambers comprising 27 columns of 10 serial chambers (Figure 1A). Each column constitutes a distinct composition of exogenous factors, whereas under continuous fluid flow, secreted paracrine factors are able to accumulate along the dimension of the serial chambers (rows 1 through 10), allowing for subsequent visualisation of differential effects on cell phenotype.

Arrays can be coated with various attachment substrata and sustain hESCs under medium perfusion for at least 7 days. Under the nominal flow conditions which we validated previously for hESCs [14], cells are exposed to creeping laminar flow and low shear stresses, while 250 µm wide interconnects spatially discretise serial chambers (computational fluid dynamic modelling, Figure 1C). Table S1 details the physical parameters and flow conditions in the microbioreactor array. Different flow requirements for preliminary and endpoint operations such as surface coating, cell seeding, and immunofluorescence labelling were realised through alternative fluidic inlets/outlets that were plugged or opened to switch between operation modes. Microbioreactor arrays had a culture area of 2.08 mm^2^ per chamber, with 2.25 mm horizontal and vertical pitch between adjacent chambers, reflecting the layout of a 1536-well microtiter plate.

To evaluate the microfluidic network performance and for visualisation and qualitative validation of the spectrum of culture conditions formed by the array, we performed dye-loading experiments and fluorimetric quantification of soluble factor levels. Colorimetric validation confirmed the successful generation of an array of discrete microenvironments within the device (Figure 1D,E), with the expected partitioning of dyes and uniform appearance along serial chambers in a column. Fluorimetric quantification of 40 kDa FITC-dextran concentrations in the array revealed clear generation of the full spectrum of designed microenvironmental compositions as well as sufficient diffusive mixing capacity (Figure 1F). Detection of low-level fluorescence in zero-concentration conditions in Channels B and C was due to residual adsorbed dye. The accurate generation of all concentration levels also implied the flow distribution across the device was in agreement with design flowrates and therefore equal between columns. Cell seeding uniformity was also evaluated using fixed, Hoechst-labelled cells and image analysis to count nuclei, which showed acceptably uniform cell distribution throughout the array (coefficient of variation 10.7%, Figure 1G). Cell attachment was also assessed by seeding live hESCs into arrays, which revealed that hESCs were attached 2 h after seeding and were uniformly distributed within a chamber (Figure 1H).

### Microbioreactor Array Screening of BMP, Activin and Wnt Signals in hESC Mesendodermal Differentiation Uncovers Complex “Paracrine Signatures”

**Figure 2:**
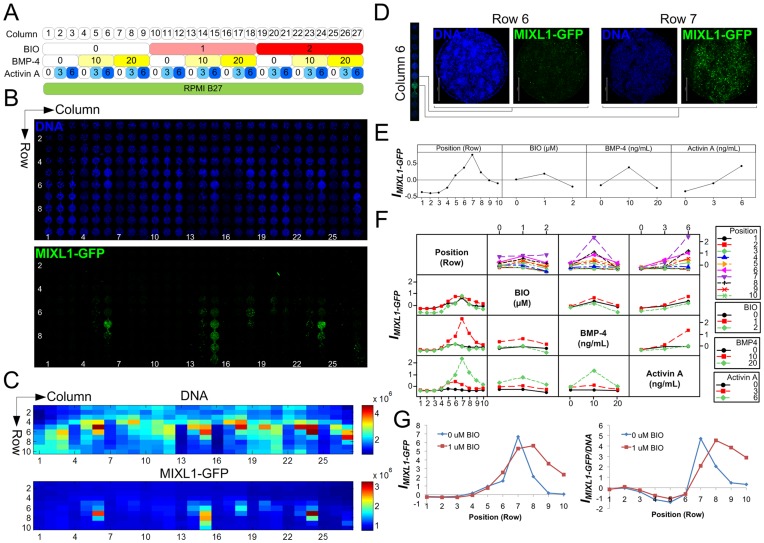
Microbioreactor screening of HES3(*MIXL1^GFP/w^*) cells undergoing mesendodermal differentiation demonstrates paracrine dependencies. **A** Screening panel showing array conditions in each column. Numbers indicate concentrations of BIO (µM), BMP-4 (ng/mL) and Activin A (ng/mL). **B** Confocal images of HES3(*MIXL1^GFP/w^*) hESCs expressing GFP and counterstained with Hoechst at experiment endpoint. Medium flow direction was from top to bottom. **C** Heat maps of total fluorescence intensities in the array (arbitrary units). **D** Higher-magnification confocal images of individual wells within the microbioreactor array highlighting position-dependent phenotypes, scale bar: 500 µm. **E** Main effect magnitudes of exogenous factors (BIO, BMP-4, Activin A) and positional dependency (Position) on expression index *I_MIXL1-GFP_*. Units represent global standard deviations relative to global mean. **F** Interaction effect magnitudes of combinations of 2 factors on expression index *I_MIXL1-GFP_*. Full dataset is shown in Figures S2, S3. **G** Traces of expression index *I_MIXL1-GFP_* and DNA-normalised expression index *I_MIXL1-GFP/DNA_* versus position coordinate for 0 (Column 6) and 1 µM BIO (Column 15) showing extended expression under BIO treatment.

To exemplify the microbioreactor array’s ability to screen and dissect extracellular patterning signals, we differentiated hESCs towards a primitive streak-like (mesendodermal) phenotype, which is marked by the transcription factor MIXL1 and known to be induced by BMP, Activin and Wnt signals [7], [15]. As a readout of MIXL1 expression status, we utilised HES3(*MIXL1^GFP/w^*) hESCs [15] (karyotyping and *in vivo* teratoma formation data, Figure S1). This reporter line contains an eGFP expression cassette inserted at the *MIXL1* locus. HES3(*MIXL1^GFP/w^*) hESCs were seeded into microbioreactor arrays and exposed to a full-factorial array of BMP-4, Activin A, and the GSK-3β inhibitor/canonical Wnt activator 6-bromoindirubin-3′-oxime (BIO) [16] in RPMI B27 medium for 2.5 d under continuous flow (Figure 2A).

MIXL1-GFP expression was activated under tightly delineated factor conditions, appearing at only selective positions in the array (Figure 2B,C). The highest expression was in columns 6, 15 and 24, which each contained 10 ng/mL BMP-4 and 6 ng/mL Activin A combined with 0, 1 or 2 µM BIO, respectively. Of note, high MIXL1-GFP-expressing chambers, which contain cells characterised by dim Hoechst DNA staining, were in each case immediately preceded by a “DNA-bright” chamber, characterised by clustered layers of cells and bright DNA staining (Figure 2D). These phenotypic observations recurred periodically throughout the array and suggest patterning of intermediate populations dependent on combinations of exogenous and paracrine factors. Factorial analysis (Figures S2, S3), which shows the average effect magnitude across the array for a single or combination of two factors, showed a linear, positive dependence for *I_MIXL1-GFP_* on Activin A, peak effects for BIO and BMP-4 at 1 µM and 10 ng/mL, respectively (Figure 2E), and highlighted the synergistic action of BMP-4 and Activin A at the best performing concentrations of 10 ng/mL and 6 ng/mL, respectively (Figure 2F).

Most notably, the dependence of MIXL1-GFP expression on Position coordinate (the row number within a series of 10 chambers) showed a peak at row 7 which was preceded by an increasing exponential trend and immediately followed by a decreasing exponential trend in MIXL1-GFP levels (Figure 2E). Across the whole array, appreciable MIXL1-GFP expression was only detected below the 4th row of serial chambers, suggesting that combinations of only BMP, Activin and canonical Wnt stimulation were not sufficient to directly activate robust MIXL1 expression at concentrations tested. Rather, this data implies that progressive accumulation of surplus, diffusible paracrine factors is required for MIXL1 activation.

Subsequent to MIXL1-GFP activation, expression intensity was attenuated rapidly in downstream chambers, suggesting presence of a negative feedback mechanism to the MIXL1-GFP^+^ population, perhaps mediated by soluble factors (Figure 2D,G). However, in column 15, which contained 1 µM BIO, bright MIXL1-GFP expression extended over 4–5 rows, rather than the single peak of fluorescence seen at row 7 in column 6, which comprised identical conditions but lacked BIO (Figure 2B,C,G). Tracing of MIXL1-GFP expression through these columns highlighted extended expression under the condition with 1 µM BIO, both in terms of absolute and DNA-normalised MIXL1-GFP expression (Figure 2G). This suggested that the negative paracrine feedback effect could potentially be overcome by enforcing canonical Wnt signalling using a GSK-3β inhibitor. Although column 24, which contained 2 µM BIO, did not have noticeably extended MIXL1-GFP expression, BIO is known to be a non-specific GSK-3β inhibitor, and is also cytotoxic at higher concentrations and lower cell densities. We therefore switched to a more specific and less cytotoxic GSK-3β inhibitor, CHIR99021 [17], to reconfirm the effect in subsequent experiments.

Such position-dependent patterns of MIXL1-GFP expression under continuous flow conditions could therefore be interpreted as representing a “paracrine signature” present under the various exogenous factor conditions, encapsulating information about the requirements for factor accumulation or presence of negative feedback loops.

Replicate arrays had Pearson’s correlation coefficients of 0.66 and 0.69 for *I_DNA_* and *I_MIXL1-GFP_*, respectively, based on paired fluorescence values of corresponding chambers (Figure S4), comparing favourably to ECM protein array experiments, which had correlation coefficients of 0.35–0.65 for averaged responses taken from a subset of spots [18]. To ensure that the array results were not an artefact of transient MIXL1-GFP induction, we also analysed MIXL1-GFP expression in an array run for 3.5 d, and found that this had a similar GFP distribution to arrays run for 2.5 days, demonstrating that absence of MIXL-GFP expression in the initial rows was not simply due to insufficient induction time or transient MIXL1 expression (Figure S5).

### Provision of Conditioned Medium and Modulation of Wnt Signalling Affect Paracrine Signatures in MIXL1-GFP Induction

Based on the emergent position-dependent expression patterns from the initial screen, which could be interpreted as “paracrine signatures”, we next hypothesised that modulation of such signatures resulting from various exogenous factor treatments would be an effective strategy to confirm the presence of putative paracrine effects and provide clues as to the identity of factors involved. In this way, direct provision of putative factors, inhibition of their associated signalling pathways, or depletion of putative factors from the medium could be applied whilst looking for changes to the expression pattern of phenotype induction.

**Figure 3:**
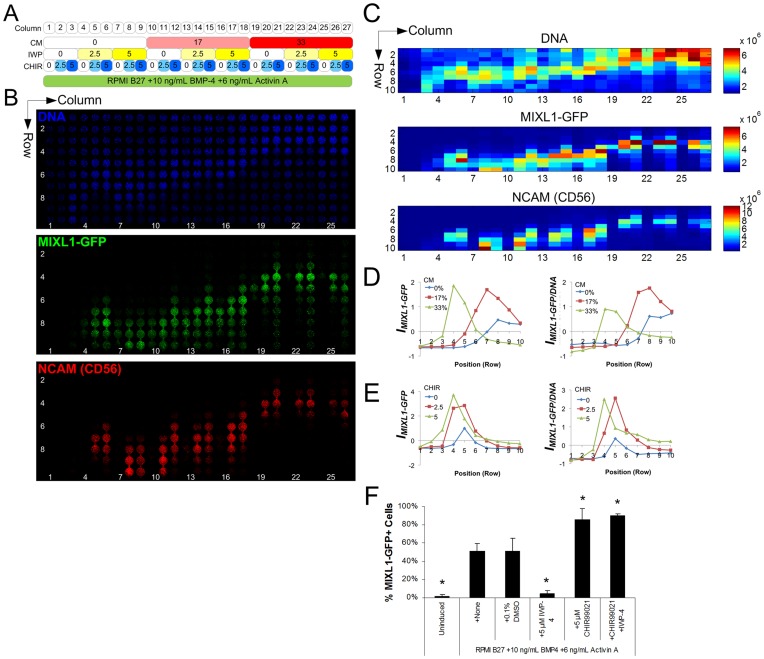
Confirmation and screening of paracrine factors involved in hESC mesendodermal differentiation. A Screening panel showing array conditions, corresponding to B-C. Numbers indicate concentrations of induction-conditioned and factor re-supplemented medium (CM, final % v/v), IWP-4 (µM), and CHIR99021 (CHIR, µM). **B** Confocal images of HES3(*MIXL1^GFP/w^*) hESCs expressing GFP and co-immunostained *in situ* for NCAM (CD56) at experiment endpoint. Flow direction was from top to bottom. **C** Heat maps of total fluorescence intensities in the array (arbitrary units). **D** Selected interaction effect plot showing average effect magnitudes for combinations of CM and Position on expression index *I_MIXL1-GFP_* and DNA-normalised expression index *I_MIXL1-GFP/DNA_*, highlighting the shift towards earlier rows with CM treatment (legend represents CM concentration in % v/v). Full dataset, Figures S6, S7. **E** Traces of expression index *I_MIXL1-GFP_* and DNA-normalised expression index *I_MIXL1-GFP/DNA_* versus position coordinate for 0 (Column 19), 2.5 (Column 20), and 5 µM CHIR (Column 21) showing extended expression under CHIR treatment. **F** Blocking of MIXL1-GFP induction in static cultures with IWP-4, and improvement and rescue of induction with CHIR. Bars represent mean ± SD of 4 biological replicates over 2 independent experiments. * indicates p<0.05 relative to +None condition.

To evaluate this in the case of MIXL1-GFP induction, we collected induction-conditioned medium (re-supplemented with 50% of nominal levels of BMP-4 and Activin A, referred to as CM) from static 2D cultures of HES3(*MIXL1^GFP/w^*) hESCs induced to differentiate towards mesendoderm, assuming that this would contain paracrine factors secreted by the cells during differentiation and thus should counteract the need for accumulation of further factors under continuous medium flow. We then constructed another screen where the whole of the array was provided with a background of the best-performing concentrations of BMP-4 and Activin A from the initial screen (10 ng/mL and 6 ng/mL respectively; Figure 3A), conditions which clearly displayed the paracrine-dependent expression pattern. CM was then directly provided as a factor in this array at 0, 17 and 33% v/v, again for 2.5 d under continuous medium flow. Indeed, in strong support of the existence of transferable paracrine factors necessary for MIXL1-GFP induction in this format, supply of CM shifted MIXL1-GFP expression towards the initial rows of the array in a dose-dependent manner (Figure 3B-D; complete factorial analysis dataset, Figures S6, S7). This time, arrays were also immuno-stained *in situ* for the early mesodermal marker NCAM [19] (also know as CD56; Figure 3B,C). NCAM also co-localised with MIXL1 expression across the array, providing further confirmation independently of the gene reporter of PS induction and a shift resulting from CM treatment. Although Column 1 did not present a clear peak at Row 7 as in the initial screen, this may be due to slight variations in cell density, paracrine output, or changes in maximum MIXL1-GFP intensity (and therefore resultant imaging parameters) in the array when CHIR was included as a factor. Nevertheless, positional shifts resulting from various factor treatments were clearly discernible.

To test the additional hypothesis arising from the initial screen – that GSK-3β inhibitors increased the extent of MIXL1-GFP expression – HES3(*MIXL1^GFP/w^*) hESCs were subjected to treatment with a distinct, highly specific GSK-3β inhibitor, CHIR99021 [17]. Similar to BIO, CHIR increased the length of chambers over which MIXL1-GFP expression persisted (Figure 3B,C,E; complete factorial analysis dataset, Figures S6, S7). We then tested whether the outcomes of modulating paracrine regulators in the array could be translated to improve directed differentiation protocols in conventional static cultures. Indeed, we found that addition of CHIR resulted in improved outcomes not only in the microbioreactor array, but also in static controls. Relative to standard conditions of RPMI B27+10 ng/mL BMP-4+6 ng/mL Activin A, addition of CHIR increased MIXL1-GFP induction from 51% to 86% of cells (Figure 3F), demonstrating applicability of array outcomes when translated to static cultures.

Regulation of the primitive streak-like phenotype by Wnt is a mechanism that has been highlighted in the literature, with several studies reporting that active Wnt signalling is required for induction of primitive streak [7], [20]. Since Wnt proteins may be involved in the effect of CM, we took a chemical stimulation and inhibition approach to evaluate Wnt pathway involvement. To assess whether Wnt is a paracrine factor required for MIXL1-GFP induction we used the small molecule inhibitor of Wnt production IWP-4 [21]. IWP-4 completely blocked MIXL1-GFP induction in static controls (Figure 3F), and did not show appreciable induction when applied over an inductive BMP/Activin background in the microbioreactor array (Figure 3B,C, Columns 4 & 7), however this could be overcome by addition of CM or CHIR (Figure 3B,C, Columns 16 & 9, respectively). Since it is expected that conditioned medium would contain Wnt proteins, and IWP-4 blocks only endogenous secretion and not exogenous factor activity, Wnt proteins present in CM or CHIR treatment could override IWP-4. Furthermore, in the absence of CM, increasing IWP-4 concentration shifted the peak of MIXL-GFP expression downstream in the array (Figure 3B,C, Columns 4–9). This indicated, in agreement with the previous reports, that Wnt stimulation is also required, and likely as a result of endogenous Wnt production. Further support for this assertion was provided by the observation that addition of CHIR could rescue the inhibitory effect of IWP-4 in static cultures (Figure 3F).

To confirm the expression of these putative inductive and inhibitory paracrine factors in the respective populations present, we sorted GFP^-^ and induced GFP^+^ cells from induced static cultures and compared their gene expression profiles with uninduced cells (Figure S8). We detected upregulation of transcripts for the canonical Wnts *WNT3A* and *WNT8A* following induction in static cultures and likewise detected upregulation of *DKK1*, a secreted Wnt antagonist and known canonical Wnt target gene [22], (Figure S8).

### FGF Stimulation Promotes the Paracrine Factor-Dependent Emergence of MIXL1-GFP^+^ Cells

**Figure 4:**
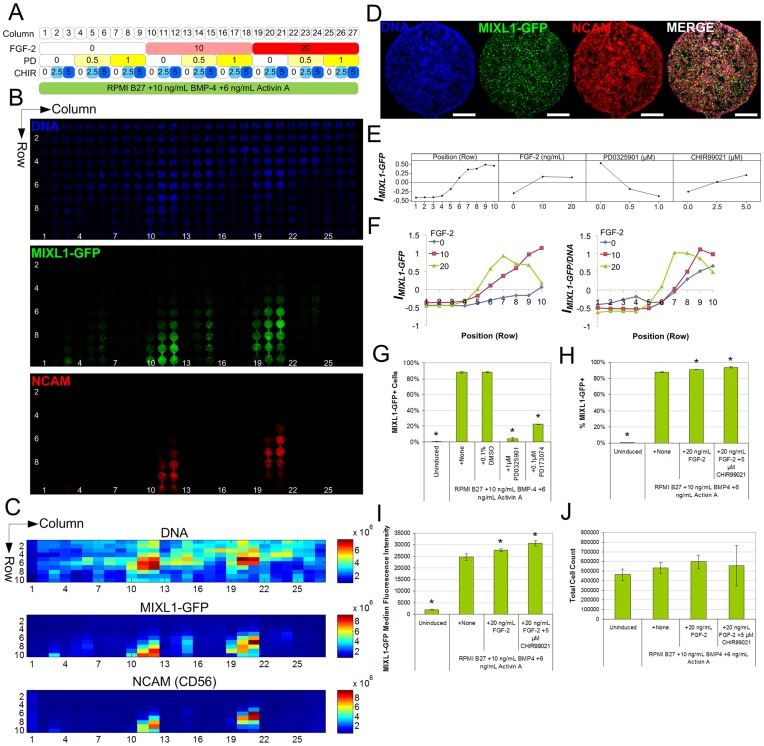
Interrogation of FGF stimulation and MEK inhibition on hESC mesendodermal differentiation in HES3(*MIXL1^GFP/w^*) hESCs. **A** Screening panel showing array conditions. Numbers indicate concentrations of FGF-2 (ng/mL), PD0325901 (PD; µM), and CHIR99021 (CHIR; µM). **B** Confocal tile scan image of HES3(*MIXL1^GFP/w^*) hESCs expressing GFP and and co-immunostained *in situ* for NCAM (CD56) and Hoechst at 2.5 d experiment endpoint. Flow direction was from top to bottom. **C** Heatmaps of total fluorescence intensities in the array (arbitrary units). **D** Higher-magnification images of cells in highest MIXL1-expressing conditions – Column 21, Row 7– demonstrating efficient induction of MIXL1-GFP and NCAM. Scale bar: 500 µm. **E** Main effect magnitudes of factors on expression index *I_MIXL1-GFP_*. Units represent global standard deviations relative to global mean. **F** Selected interaction effect plots showing average effect magnitudes for combinations of FGF-2 and Position on expression index *I_MIXL1-GFP_* and DNA-normalised expression index *I_MIXL1-GFP/DNA_*, highlighting the shift towards earlier rows with FGF-2 treatment (legend represents FGF-2 concentration in ng/mL). **G** Chemical inhibition of MIXL1-GFP induction with MEK cascade inhibitor PD0325901 and FGF receptor tyrosine kinase inhibitor PD173074. Bars represent mean ± s.d., *n* = 2−3 from one representative experiment. **H-J** Addition of FGF-2 or FGF-2+ CHIR99021 improved induction efficiency (**H**) and median fluorescence intensity (**I**) in static controls, but without significantly increasing total cell count (**J**). Bars represent mean ± s.d., *n* = 4 from 2 independent experiments. * *p*<0.05 compared to +None condition, one-way ANOVA.

While Wnt was identified as a critical component of the paracrine inducing factors, induction of MIXL1-GFP did not proceed from the top rows of the array when treated with CHIR, suggesting that other types of factors may also be required. Based on FGF’s proposed role in mesodermal induction [23], and to demonstrate the array’s capability for screening specific paracrine signals, we investigated stimulation of FGF signalling by screening FGF-2 as a potential paracrine factor, as well as an inhibitor of the MEK signalling cascade downstream of FGF. HES3(*MIXL1^GFP/w^*) hESCs were grown under the same background of RPMI B27/BMP-4/Activin A and subjected to stimulation under continuous flow for 2.5 d with FGF-2, the MEK cascade inhibitor PD0325901, and CHIR (Figure 4A). The array identified FGF-2 and CHIR as having a positive effect on induction, whereas PD0325901 strongly inhibited MIXL1-GFP expression (Figure 4B,C,E). FGF-2 acted similarly to conditioned medium in shifting MIXL1-GFP expression towards initial rows of the array in a dose-dependent manner (Figure 4B,C,F), whereas the MEK cascade inhibitor PD0325901 completely blocked MIXL1 induction in the array. FGF-2 in combination with BMP, Activin, and Wnt signals was efficient in inducing MIXL1-GFP^+^ and NCAM^+^ cells in the highest-expressing chamber (Figure 4D). PD0325901 as well as the FGF receptor tyrosine kinase inhibitor PD173074 strongly inhibited MIXL1-GFP induction in static controls (Figure 4G), suggesting FGFs are endogenously accumulated paracrine components involved in MIXL1 activation. In static controls, addition of FGF-2 in combination with CHIR further increased the percentage of positive cells (Figure 4H) and median fluorescence intensity (Figure 4I), without significantly increasing total cell number (Figure 4J), suggesting the effect did not solely arise from mitogenic effects. This again highlighted the transferability of array results to improving outcomes of static culture protocols.

## Discussion

During development, complex stimuli lead to progenitor cell specification, differentiation, and patterning to form the embryo. One of the earliest patterning processes in the embryo is primitive streak formation, a transient structure marked by the transcription factor MIXL1. Analysis of mouse embryonic development using knock-out models, local delivery of patterning molecules and whole mount *in situ* hybridization support the current view that exposure of embryonic stem cells migrating through primitive streak to dynamic gradients of BMP, Activin and Wnt signals as well as cell-cell contact cues pattern cells into progenitors of the heart, kidney and blood lineages [24], [25]. Primitive streak formation in humans is thought to proceed in an analogous fashion to the mouse, although this has been difficult to investigate to date, due to ethical constraints. Exposure of hESC cultures to different BMP, Activin and Wnt molecule regimes indeed induces primitive streak formation and this is widely used as the initial patterning process for generating tissue specific progenitors and subsequently fully differentiated cell types. However, even the most carefully optimised PS induction protocols do not generate homogeneously patterned progenitor cell populations, suggesting that either the starting cell population is heterogeneous or that the patterning molecules do not solely act in a cell autonomous fashion, but also trigger the release of positively and negatively acting paracrine signalling molecules. Previous work which used factor-treated “stimulator” mESCs forcibly aggregated with Mixl1*^GFP/w^* reporter gene-marked, “responder” mESCs in the absence of inducing factors was able to demonstrate PS induction in the responder cells when the stimulator cells were treated with BMP4 or Wnt3a, suggesting these induce paracrine factors which are then able to induce reporter expression [7]. Blockade of Wnt or Activin signalling at the time of aggregation was also able to block reporter induction, in line with the results obtained in this work using the Wnt inhibitor IWP-4.

If human pluripotent stem cell based regenerative medicine is to succeed, significant numbers defined lineage progenitors will need to be derived in an efficient and homogeneous fashion. Identification and judicious modulation of endogenously produced paracrine factors or their signalling pathways would provide a means to achieve this aim. Current culture methodologies, such as standard 2D culture systems and 3D embryoid body-based differentiation strategies, do not readily lend themselves to identification of such paracrine factors, and do not provide a static, defined culture environment due to the accumulation and depletion of such factors and metabolites over time. To overcome these hurdles we have designed microbioreactor arrays capable of dissecting and visualising paracrine signalling effects simultaneously with full factorial exogenous factor provision. Remarkably, we find that, at appropriate cell densities and factor concentrations, induction of a primitive streak-like population by BMP-4, Activin A and BIO (a small molecule Wnt activator) is reliant on accumulation of surplus paracrine factors, as indicated by the specific emergence of MIXL1-GFP expression in downstream serially connected microfluidic chambers, and not in the initial chambers, and that this is not a function of temporal expression of MIXL1 in specific subpopulations.

The presence of such diffusible, paracrine factors is most convincingly exemplified by the fact that we are able to shift MIXL1-GFP expression towards the top of the array by applying conditioned medium from PS-induced hESC cultures. Furthermore, we are able to recapitulate this phenomenon through the addition of FGF-2, suggesting FGF-2 or an analogous family member with similar biological activity is one of the paracrine factors produced by the PS-inducing growth factor regimen. Nevertheless, supplementation of BMP, Activin, FGF and canonical Wnt signals at concentrations tested was not sufficient to initiate expression from the very top of the array, signifying that they are limiting or that additional factors are involved, for instance, they may stimulate secretion of Nodal, which has Activin-like activity and is also induced by Activin. Alternatively, metabolic products [26], co-receptors/factors (e.g. HSPGs), extracellular matrix components or proteases/protease inhibitors may be required. Our microbioreactor system provides a unique screening platform to now identify such factors at reasonable throughput.

Subsequent to MIXL1-GFP activation, expression intensity was attenuated rapidly in downstream culture chambers, suggesting that the MIXL1-GFP^+^ population is regulated by a narrow concentration range of diffusible factors, or itself produces inhibitory factors which oppose its expression or promote exit from a transient MIXL1-GFP^+^ state. Enforced Wnt activation by the GSK-3β inhibitors BIO and CHIR suggest that a negative feedback mechanism mediated by Wnt inhibitory factors is responsible. Indeed, RT-qPCR analysis revealed *T*, *MIXL1*, *WNT3A, WNT8A* and *DKK1* expression in hESCs induced to form PS (Figure S8). Strong upregulation of *WNT8A* was detected in induced cells in this study, in agreement with previous studies on *WNT8A* homologues showing that *wnt8a* is able to induce a complete secondary axis in *Xenopus laevis* [27] embryos, and *Wnt8a* is expressed in mouse primitive streak [28]. *DKK1* was expressed 13-fold higher in MIXL1-GFP^+^ cells than in uninduced cells, in concordance with previous studies showing that positive (*WNT3A*) and negative (*DKK1, SFRPs*) extracellular regulators of canonical Wnt signalling are transiently expressed through 3–4 d in hESCs differentiating towards the cardiac lineage [29], [30] and coincides with the timing of expression of primitive streak markers *MIXL1* and *T*. It has further been reported that Wnt3a-stimulated mouse ES cells form *T* ^+^ PS and activate a negative feedback loop mediated by Dkk-1 [31]. Since BIO and CHIR act intracellularly at GSK-3β to activate β-catenin-mediated canonical Wnt signalling, their effect should be immune to the inhibitory effect of paracrine DKK1 (which binds to the Wnt co-receptor LRP5/6) or SFRPs (which sequester WNTs), and this was indeed supported by the microbioreactor array data.

Altogether, the results presented highlight that our microbioreactor array platform is not only able to replicate many known facets of primitive streak induction commonly observed in standard culture formats, but this unique device platform is able to generate outcomes and insights into cell signalling that can only be observed in such serially-connected, perfused microenvironments. Further, the data confirms that this device can then be used to improve the efficiency of directed differentiation protocols in static cultures. The microbioreactor array is clearly a useful tool to examine and modulate paracrine effects, providing strong visual evidence of shifts in “paracrine signatures” and allows hypotheses to be made and tested regarding the presence of paracrine factors through the inclusion of putative factors, inhibitors, siRNAs or inactivating antibodies. The inclusion of multiple factor channels in the microbioreactor array is critical in allowing side-by-side comparisons of the effects of introducing putative paracrine inducing factors or chemical inhibitors of their action and provides an assay platform for directly assessing the impact of supplemented factors and the hierarchy of their actions. Identification and supplementation of positively-acting factors and inhibition of negatively-acting factors can then be used to maximise differentiation outcomes towards defined phenotypes, as shown in this paper.

### Conclusions

This paper applied a microbioreactor array platform multiplexing exogenous and paracrine factors to hESC differentiation to a primitive streak-like population. Paracrine effects are extremely difficult to assess in static cultures, and we demonstrate here that these microbioreactor arrays are very useful in the study of their effects in controlling cell fate. By revealing paracrine signatures that result from various exogenous factor stimulation regimes, the device provided an assay to identify agents that modulate paracrine-dependent outcomes, and thereby allowed decoding of the hierarchy of direct-acting inducing factors as well as inhibitory, negative feedback factors. Importantly, these results were then translated to improve directed differentiation protocols in static cultures. Using the microbioreactor array platform we demonstrated that mapping of combinations of both exogenous and paracrine stimulation is possible and therefore use of this technology is generally applicable to studies examining the effect of external stimuli on cell behaviour and phenotype.

## Methods

All reagents were obtained from Sigma-Aldrich unless otherwise mentioned.

### Microbioreactor Design, Fabrication and Validation

Microbioreactor arrays were designed using scalable, hierarchically-nested, resistive flow-based dilution networks, as described previously [13]. Array designs were fabricated by SU-8 2100 photolithography and poly(dimethylsiloxane) (PDMS) soft lithography [32], and were assembled as described previously [13]. For visualisation and qualitative validation of conditions, red, yellow and blue food dyes and PBS buffers were perfused through the device at 60 µL/h total flowrate. Solution concentrations in each column were quantitatively validated using a 40 kDa (average MW) FITC-dextran solution (100 µM in PBS). FITC-dextran solution was perfused through each of the factor inlets (A1, B1, C1) and quantified independently and serially, with all other inlets containing PBS. Fluorescence levels were quantified by fluorescence microscopy and compared against known standards prepared by micropipetting and imaged in similar microbioreactor chambers. Solution concentration was related to fluorescence intensity by a linear function with *R* ^2^>0.99 in the concentration range of interest.

### Cell Culture and Static Controls

Human embryonic stem cells were dealt with under approval of the UQ Medical Research Ethics Committee, and were maintained by and obtained from StemCore, the Australian Stem Cell Centre’s core hESC laboratory. HES3(*MIXL1^GFP/w^*) hESCs [15] were used between passages 79 and 86, and were cultured in mTeSR-1/Matrigel cultures for 1–3 passages before array experiments. Cells were verified for a normal karyotype (passage 86) and teratoma formation capacity (passage 77), as shown in Figure S1. Static culture controls in 24-well plates were coated at solution concentrations adjusted to supply equivalent total amounts of protein per surface area, and were also seeded with equivalent surface densities of cells. HES3(*MIXL1^GFP/w^*) hESCs maintained in mTeSR-1/Matrigel cultures were detached with TrypLE Express, seeded at 7.5×10^4^ cells/cm^2^ into 24-well plates coated with a limiting dilution of hESC-qualified Matrigel and allowed to attach overnight, after which cultures were typically ∼70% confluent. MIXL1-GFP expression was then induced by differentiating in RPMI B27 medium with 10 ng/ml BMP-4 and 6 ng/ml Activin A (both R&D Systems) for 2.5 d, unless indicated otherwise. Additional small molecules or factors were optionally included at the start of induction at concentrations indicated: IWP-4, an inhibitor of Wnt production and signalling [21]; CHIR99021, a specific GSK-3β inhibitor/canonical Wnt activator; PD0325901, a MEK cascade inhibitor; PD173074, an FGF receptor tyrosine kinase inhibitor (all Stemgent); or FGF-2 (Millipore). 0.1% v/v DMSO was used as a vehicle control.

### Microbioreactor Array Screening

Microbioreactor arrays were autoclaved, filled with sterile PBS by the channel-outgas technique [33], then surface-coated (2–4 h, room temperature) with a limiting dilution of hESC-qualified Matrigel (BD Biosciences). hESCs maintained in tissue culture flasks were single-cell dissociated with TrypLE Express (Gibco), then washed with and resuspended at 3.0×10^6^ cells/mL and seeded into microbioreactor arrays (7.5×10^4^ cells/cm^2^). Cells were allowed to attach for 8–10 h in an incubator (37°C, 5% CO_2_ in air), then subjected to continuous fluid flow under the specified factor conditions at 60 µL/h total flowrate. Positive-displacement-driven flow was provided by a syringe pump (NE-1800, New Era Pump Systems), *via* 3 or 1 mL syringes (Terumo), and polyethylene tubing (PE50, 0.58 mm ID, BD Biosciences), with stainless steel, 22 gauge blunt-nose needle tips as connectors.

Stock factor solutions are provided at 3× the highest concentration required in the array, to allow for subsequent dilution through diffusive mixing in the array. For HES3(*MIXL1^GFP/w^*) experiments, the stock factor/buffer pairs were: RPMI B27+6 µM BIO (Stemgent)/RPMI B27; RPMI B27+60 ng/mL BMP-4 (R&D Systems)/RPMI B27; and RPMI B27+18 ng/mL Activin A (R&D Systems)/RPMI B27. RPMI B27 medium consisted of RPMI 1640 basal medium (Gibco) +2% v/v B27 Supplement (Invitrogen). For subsequent experiments, the factor/buffer pairs were: Conditioned Medium/RPMI B27 10B6A; RPMI B27 10B6A +15 µM IWP-4 (Stemgent)/RPMI B27 10B6A; and RPMI B27 10B6A +15 µM CHIR99021 (Stemgent)/RPMI B27 10B6A, where RPMI B27 10B6A consisted of RPMI B27+10 ng/mL BMP-4, +6 ng/mL Activin A. Conditioned medium (CM) was recovered from static controls differentiated with 10 ng/mL BMP-4 and 6 ng/mL Activin A in RPMI B27 medium. Cells were removed by centrifugation and supernatant medium stored at 4°C. Media from days 1 and 2 were combined and re-supplemented with 5 ng/mL BMP-4 and 3 ng/mL Activin A (i.e. 50% of nominal levels) before use in arrays, to account for depletion and degradation. This CM was then titrated in as a factor up to 33% v/v, against base medium of RPMI B27 10B6A. For FGF-2 screening, CM and IWP-4 factors were replaced with 60 ng/mL FGF-2 (Millipore) and 3 µM PD0325901 (Stemgent), respectively. These stock factor/buffer pairs respectively resulted in the generation of conditions predicted in Figures 2A, 3A and 4A. All media were supplemented with 1% v/v penicillin/streptomycin solution (Gibco).

### *In situ* Immunofluorescence Staining and Confocal Imaging

Arrays were terminated 2.5 d after the start of fluid flow for *in situ* immunostaining. Factor/buffer inlets were plugged closed and the common seeding/coating inlet/outlet left open, with serial exchange of staining and washing solutions driven by a syringe pump. The immunodetection phase did not noticeably affect cell numbers or GFP fluorescence in the array. Arrays were washed with PBS and fixed with 2% paraformaldehyde/PBS solution (30 min, RT), followed by blocking with 3% bovine serum albumin (BSA)/PBS solution with 0.2% sodium azide (30–45 min, RT), then stained with 10 µg/mL Hoechst 33342 (Molecular Probes) in 0.3% BSA/PBS (1 h, RT). HES3(*MIXL1^GFP/w^*) arrays were stained only with Hoechst, or optionally blocked with 0.3% BSA/PBS with 0.02% sodium azide and stained with NCAM-PE (1∶10, BioLegend) along with Hoechst. Arrays were finally washed/mounted in 0.3% BSA/PBS for imaging. 16-bit, multi-colour montage images of entire arrays were acquired with a Zeiss LSM 710 laser scanning confocal microscope system and Zen 2008 acquisition software (Carl Zeiss). To adjust for intensity variations in the *z*-direction, 3 optical sections were acquired and then processed into a maximum intensity projection for image analysis. Images were linearly adjusted for publication.

### Flow Cytometry

Static control samples from 24-well plates were analysed by flow cytometry. Cells were washed with PBS, detached with TrypLE Express, neutralised with complete medium and aspirated into eppendorf tubes. Samples were centrifuged and fixed in 2% paraformaldehyde/PBS solution (30 min, RT). Cells were resuspended in wash solution and GFP expression was detected with an Accuri C6 flow cytometer. Uninduced cells were used as a negative control with the GFP expression cutoff set to give a false positive rate <1% of cells.

### Cell Sorting and Gene Expression Profiling

HES3(*MIXL1^GFP/w^*) hESCs maintained in mTeSR-1/Matrigel cultures were differentiated in RPMI B27 medium with 10 ng/ml BMP-4 and 6 ng/ml Activin A. After 2 d, cells were detached with TrypLE express, washed with mTeSR-1, then resuspended in DMEM/F12+2 µg/mL propidium iodide (Molecular Probes), and placed on ice. Viable, single cells were identified on the basis of FSC and SSC parameters and PI exclusion, then sorted for GFP^+^ and GFP^-^ fractions (top 10% and bottom 20% of target population in terms of GFP-expression, respectively) with a Cytopeia Influx instrument (BD Biosciences). Total RNA was extracted from sorted fractions and uninduced controls with RNeasy kits combined with on-column DNase treatment (Qiagen). RNA was quantified with a Nanodrop 1000 spectrophotometer (Thermo Scientific) and ∼350ng RNA used per reaction to synthesize cDNA, which was stored at −20°C until RT-qPCR was performed with Platinum SYBR Green qPCR SuperMix-UDG or Ssofast EvaGreen qPCR supermix (172–5200, Biorad). Thermocycling and analysis were performed using an ABI 7500 Fast Real-Time PCR system (Applied Biosystems) or a CFX96 Real-Time/C1000 thermal cycler (Biorad Systems) with fast cycling parameters of 50°C for 2 min (UDG incubation), 95°C for 2 min (denaturation) and then 95°C for 3 s and 60°C for 30 s for a total of 40 cycles. Results were analyzed using the 2^−ΔCt^ method relating gene expression to *GAPDH* and were further normalised to the control (uninduced) sample. Melt curve analysis was used to ensure product specificity, along with reverse transcriptase-deficient and water controls. Primer sequences (Table S2) were validated using RNA collected from experiments.

### Data Processing and Statistical Methods

Total fluorescence intensities (*T_MIXL1-GFP_*, for example) were extracted using AGScan software (www.sigenae.org). Spot intensities were linearly transformed about the mean and standard deviation for all spots in that channel in an individual array, by *I_MIXL1-GFP_* = (*T_MIXL1-GFP_ - µ_MIXL1-GFP_)*/*σ_MIXL1-GFP_*, where *I_MIXL1-GFP_* is termed the expression index of MIXL1-GFP, and *µ_MIXL1_*_-GFP_ is the mean and *σ_MIXL1_*_-GFP_ the standard deviation of all spot intensities (*T_MIXL1_*_-GFP_) for a particular array.

Factorial analyses were performed on expression index data with MINITAB 15 software (Minitab Inc.), using exogenous factor levels and row coordinate (Position) as input variables. Effect magnitudes were calculated by MINITAB as described elsewhere [34]. Pearson product-moment correlation coefficients (*r_X,Y_*) and coefficients of determination for linear regression (*R* ^2^) were calculated with Microsoft Excel. For pair wise comparisons, one-way ANOVA with post-hoc Tukey or Games-Howell tests were performed with SPSS Statistics 17.0. Differences with *p*<0.05 were considered significant. Kolmogorov-Smirnov tests were used for data normality, and Levene’s tests for homogeneity of variance.

## Supporting Information

Figure S1 Karyotyping and *in vivo* teratoma formation of HES3(*MIXL1^GFP/w^*) hESCs. (**a**) G-banding karyotyping of HES3(*MIXL1^GFP/w^*) hESCs revealed a normal human female karyotype. (**b**) Teratomas formed by HES3(*MIXL1^GFP/w^*) hESCs included elements of all three germ layers.
(TIF)

Figure S2 Factorial analysis of array data for HES3(*MIXL1^GFP/w^*) hESCs – main effects. (a-b) Main effects plots mapping effect magnitudes of the individual factors Position, BIO, BMP-4 and Activin A on *I_DNA_* (**a**) and *I_MIXL1-GFP_* (**b**) expression indices. Units are ± global standard deviations relative to global mean for each marker.
(TIF)

Figure S3 Factorial analysis of array data for HES3(*MIXL1^GFP/w^*) hESCs – interaction effects. (**a-b**) Interaction effects plots showing effect magnitudes for sets of two combined factors on expression index means for *I_DNA_* (**a**) and *I_MIXL1-GFP_* (**b**) expression indices. Units are ± global standard deviations relative to global mean for each marker.
(TIF)

Figure S4 Comparison of replicate microbioreactor array experiments. (**a**) Confocal tile scan images of HES3(*MIXL1^GFP/w^*) hESCs expressing GFP and counterstained with Hoechst 33342 at 2.5 d experiment endpoint in replicate arrays. Similar distributions of DNA and GFP intensities were observed. Maximum intensity projection of a z-sectioned image is shown, and has been linearly enhanced for publication. Raw images were used for analysis. (**b**) Heatmaps of total fluorescence intensities in replicate arrays for each marker (arbitrary units). Similar distributions of DNA and GFP intensities were observed. (**c**) Scatterplots of total spot intensities from replicate arrays. *R* ^2^ values of least-squares linear fit and Pearson’s *r* values are shown. Outliers in MIXL1-GFP intensities are marked with array position. Replicate array experiments were highly correlated.
(TIF)

Figure S5 Array run for 3.5 d. (**a**) Confocal tile scan images of HES3(*MIXL1^GFP/w^*) hESCs expressing GFP and counterstained with Hoechst 33342 at 3.5 d experiment endpoint. Similar distributions of DNA and GFP intensities were observed as for arrays run for 2.5 d. Maximum intensity projection of a z-sectioned image is shown, and has been linearly enhanced for publication. Raw images were used for analysis. (**b**) Heatmaps of total fluorescence intensities in replicate arrays for each marker (arbitrary units). Similar distributions of DNA and GFP intensities were observed as for arrays run for 2.5 d.
(TIF)

Figure S6 Factorial analysis of array data for HES3(*MIXL1^GFP/w^*) hESCs – main effects. (a-c) Main effects plots mapping effect magnitudes of the individual factors Position, CM, IWP and CHIR on *I_DNA_* (**a**), *I_MIXL1-GFP_* (**b**), and *I_NCAM_* (**c**) expression indices. Units are ± global standard deviations relative to global mean for each marker.
(TIF)

Figure S7 Factorial analysis of array data for HES3(*MIXL1^GFP/w^*) hESCs – interaction effects. (a-c) Interaction effects plots showing effect magnitudes for sets of two combined factors on expression index means for *I_DNA_* (**a**), *I_MIXL1-GFP_* (**b**), and *I_NCAM_* (**c**) expression indices. Units are ± global standard deviations relative to global mean for each marker.
(TIF)

Figure S8 Gene expression profiling of MIXL1-GFP-sorted populations. (**a**) Cell sorting strategy. Live, single cells were identified using FSC and SSC area and width parameters and propidium iodide exclusion, and then the top 10% and bottom 20% of cells in terms of GFP-expression were sorted into separate fractions. (**b**) RT-qPCR quantification of gene expression in control (uninduced, unsorted) and 2 d induced, MIXL1-GFP-sorted cell populations. Bars represent mean ± s.e.m. (*n* = 6 from 3 independent sorts) of gene expression relative to *GAPDH*, normalised to undinduced samples. * indicates *p*<0.05, one-way ANOVA. Relative to uninduced cells, induced cells showed upregulation of mesendodermal markers *T* and *MIXL1*, soluble Wnt molecules *WNT3A* and *WNT8A*, and the soluble Wnt antagonist *DKK1*.
(TIF)

Table S1 Physical Parameters.
(DOC)

Table S2 RT-qPCR Primer Sequences.
(DOC)
