# Supplementary figures and images for: Microbioreactor Arrays for Full Factorial Screening of Exogenous and Paracrine Factors in Human Embryonic Stem Cell Differentiation
Source: PLoS One. 2012 Dec 26;7(12):e52405. doi: 10.1371/journal.pone.0052405 (PMC3530582; doi:10.1371/journal.pone.0052405)

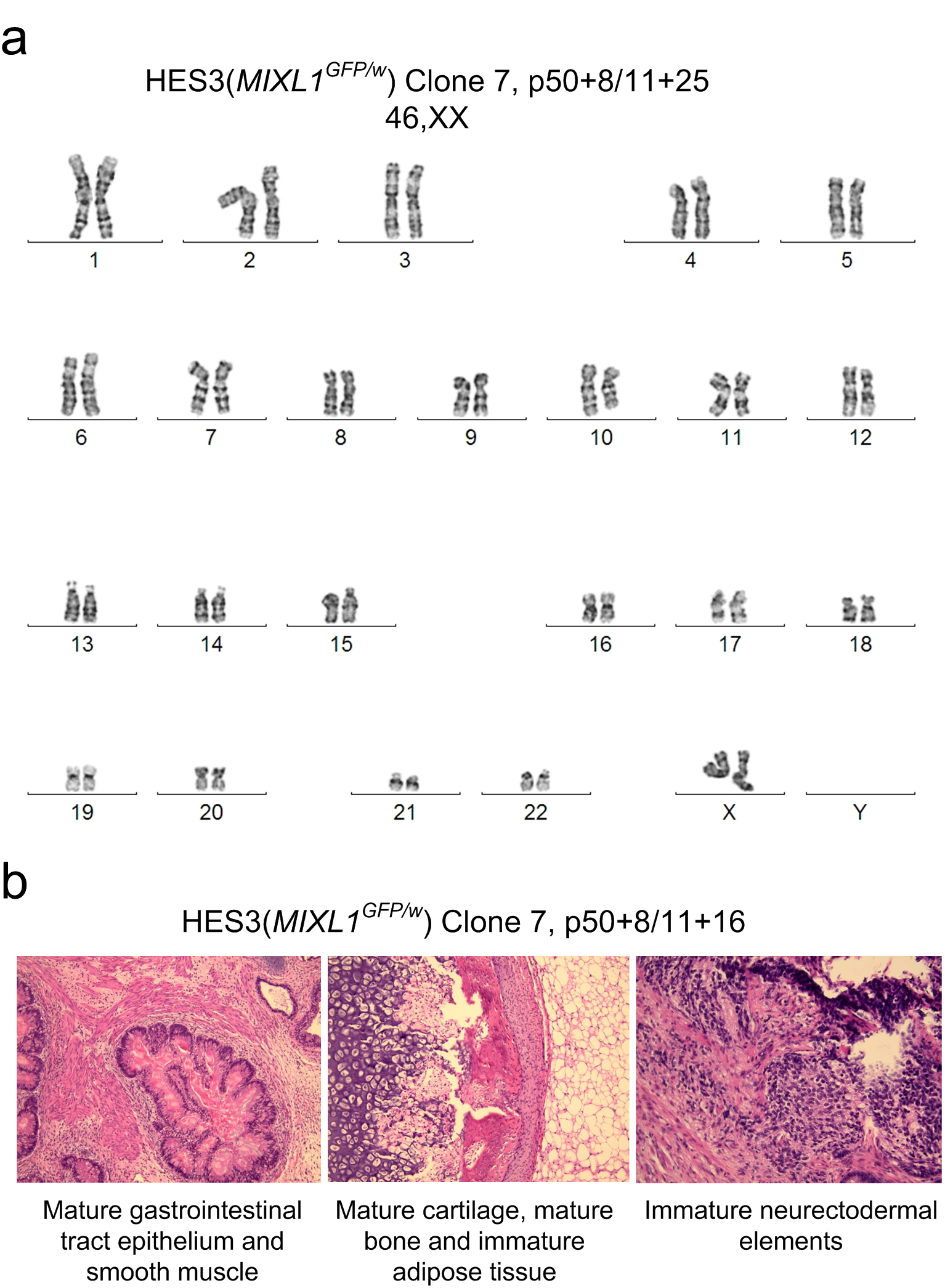

Supplement: Figure S1 — Karyotyping and in vivo teratoma formation of HES3(MIXL1GFP/w) hESCs. (a) G-banding karyotyping of HES3(MIXL1GFP/w) hESCs revealed a normal human female karyotype. (b) Teratomas formed by HES3(MIXL1GFP/w) hESCs included elements of all three germ layers. (TIF) [file pone.0052405.s001.tif]

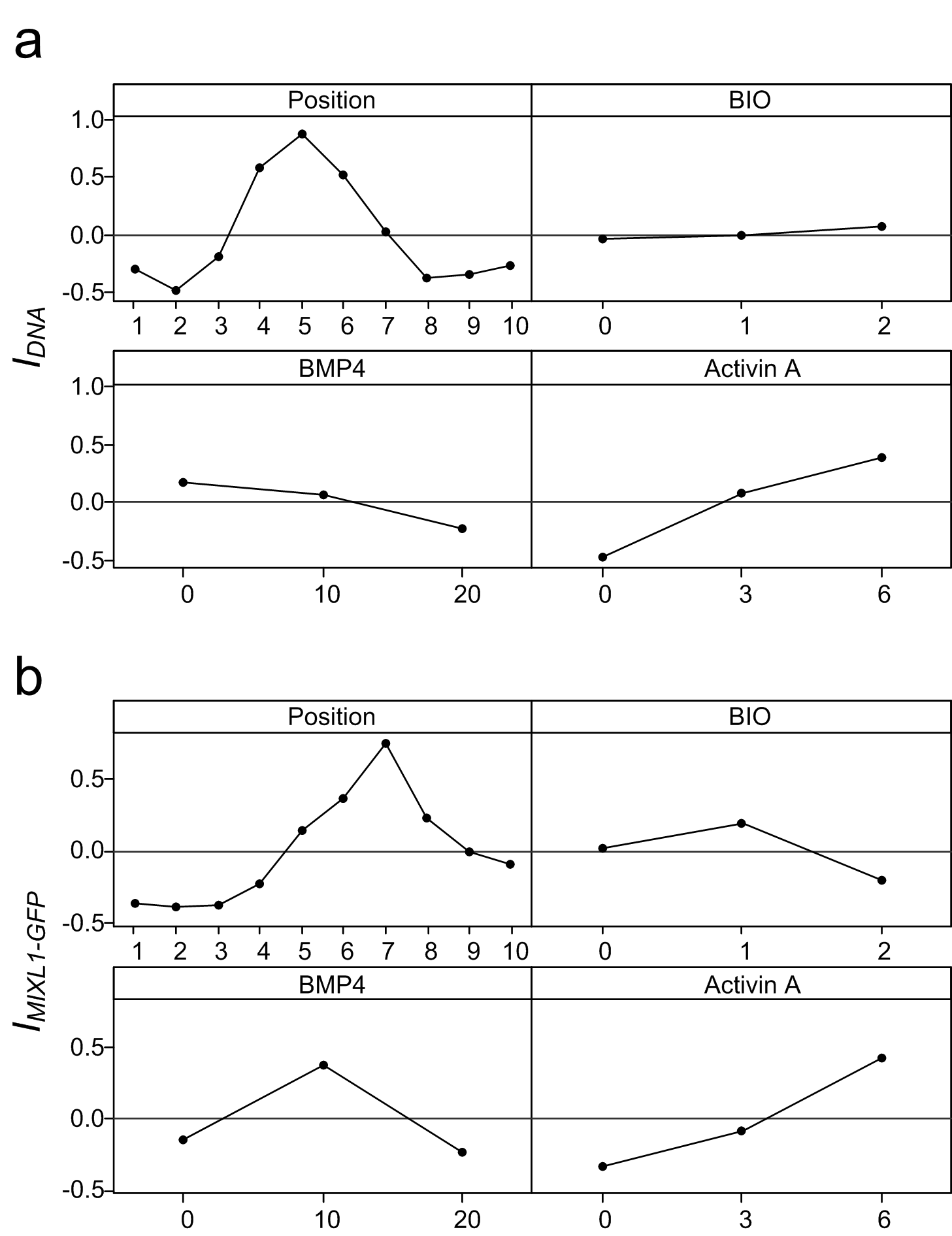

Supplement: Figure S2 — Factorial analysis of array data for HES3(MIXL1GFP/w) hESCs – main effects. (a-b) Main effects plots mapping effect magnitudes of the individual factors Position, BIO, BMP-4 and Activin A on IDNA (a) and IMIXL1-GFP (b) expression indices. Units are ± global standard deviations relative to global mean for each marker. (TIF) [file pone.0052405.s002.tif]

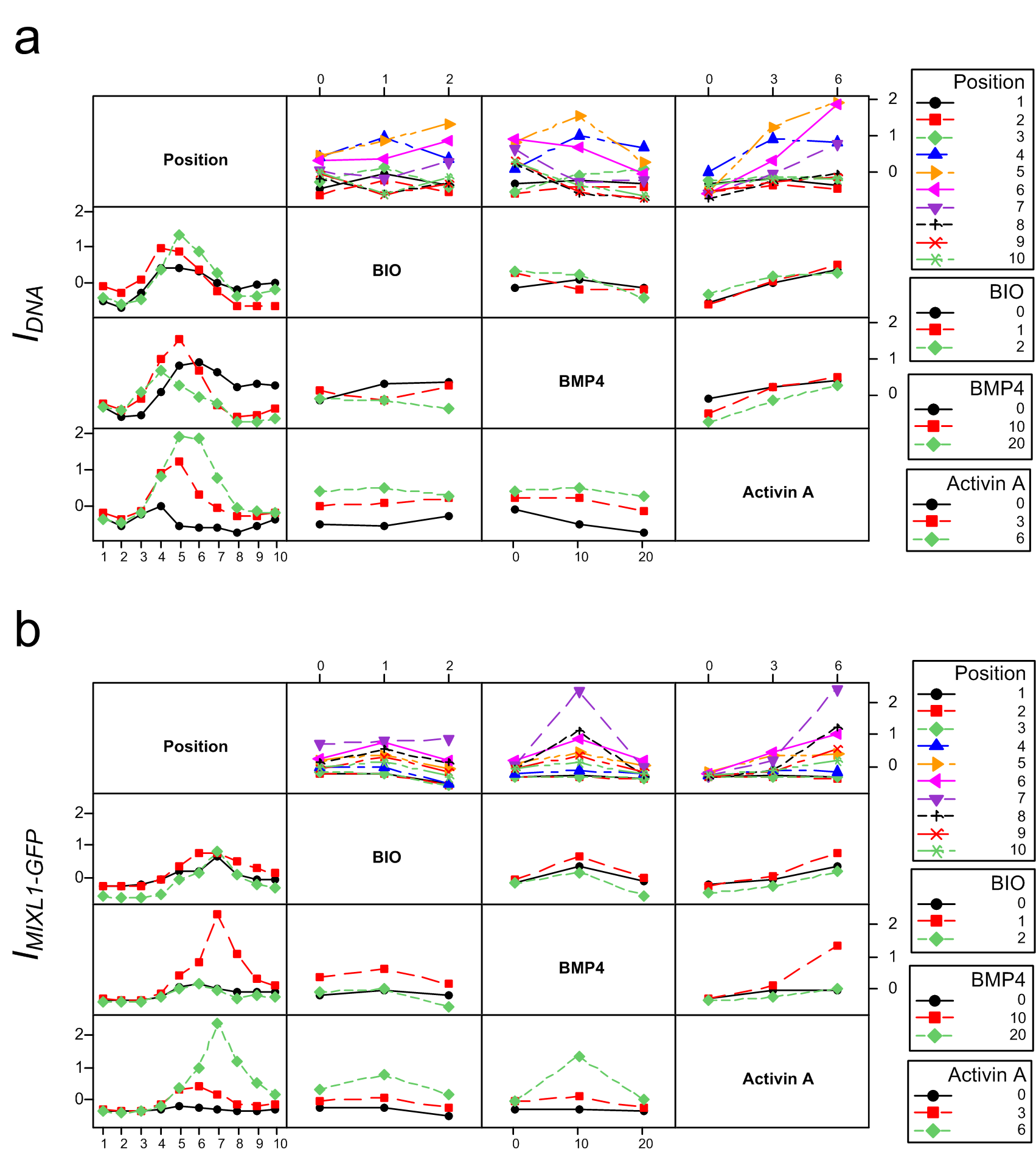

Supplement: Figure S3 — Factorial analysis of array data for HES3(MIXL1GFP/w) hESCs – interaction effects. (a-b) Interaction effects plots showing effect magnitudes for sets of two combined factors on expression index means for IDNA (a) and IMIXL1-GFP (b) expression indices. Units are ± global standard deviations relative to global mean for each marker. (TIF) [file pone.0052405.s003.tif]

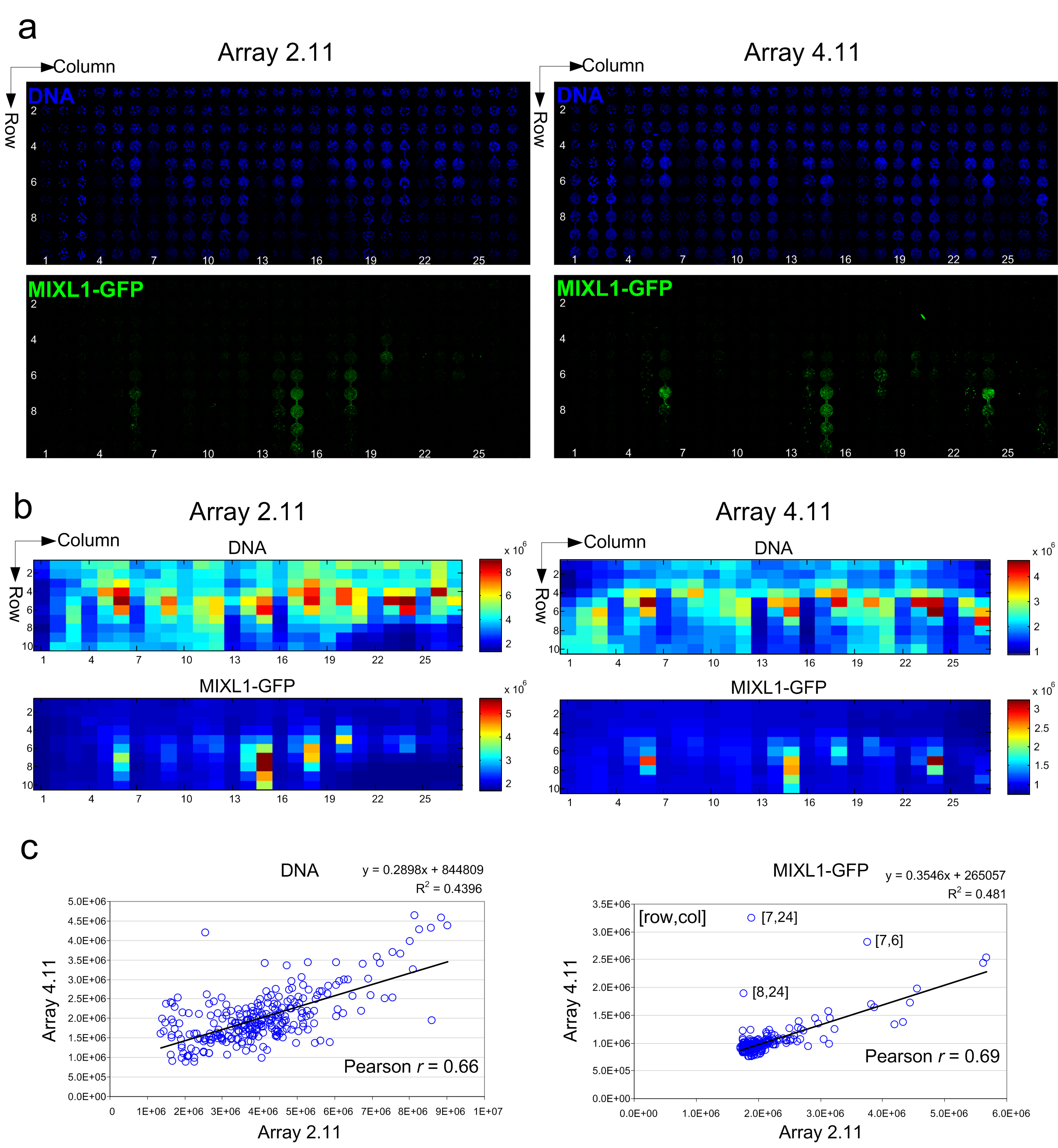

Supplement: Figure S4 — Comparison of replicate microbioreactor array experiments. (a) Confocal tile scan images of HES3(MIXL1GFP/w) hESCs expressing GFP and counterstained with Hoechst 33342 at 2.5 d experiment endpoint in replicate arrays. Similar distributions of DNA and GFP intensities were observed. Maximum intensity projection of a z-sectioned image is shown, and has been linearly enhanced for publication. Raw images were used for analysis. (b) Heatmaps of total fluorescence intensities in replicate arrays for each marker (arbitrary units). Similar distributions of DNA and GFP intensities were observed. (c) Scatterplots of total spot intensities from replicate arrays. R 2 values of least-squares linear fit and Pearson’s r values are shown. Outliers in MIXL1-GFP intensities are marked with array position. Replicate array experiments were highly correlated. (TIF) [file pone.0052405.s004.tif]

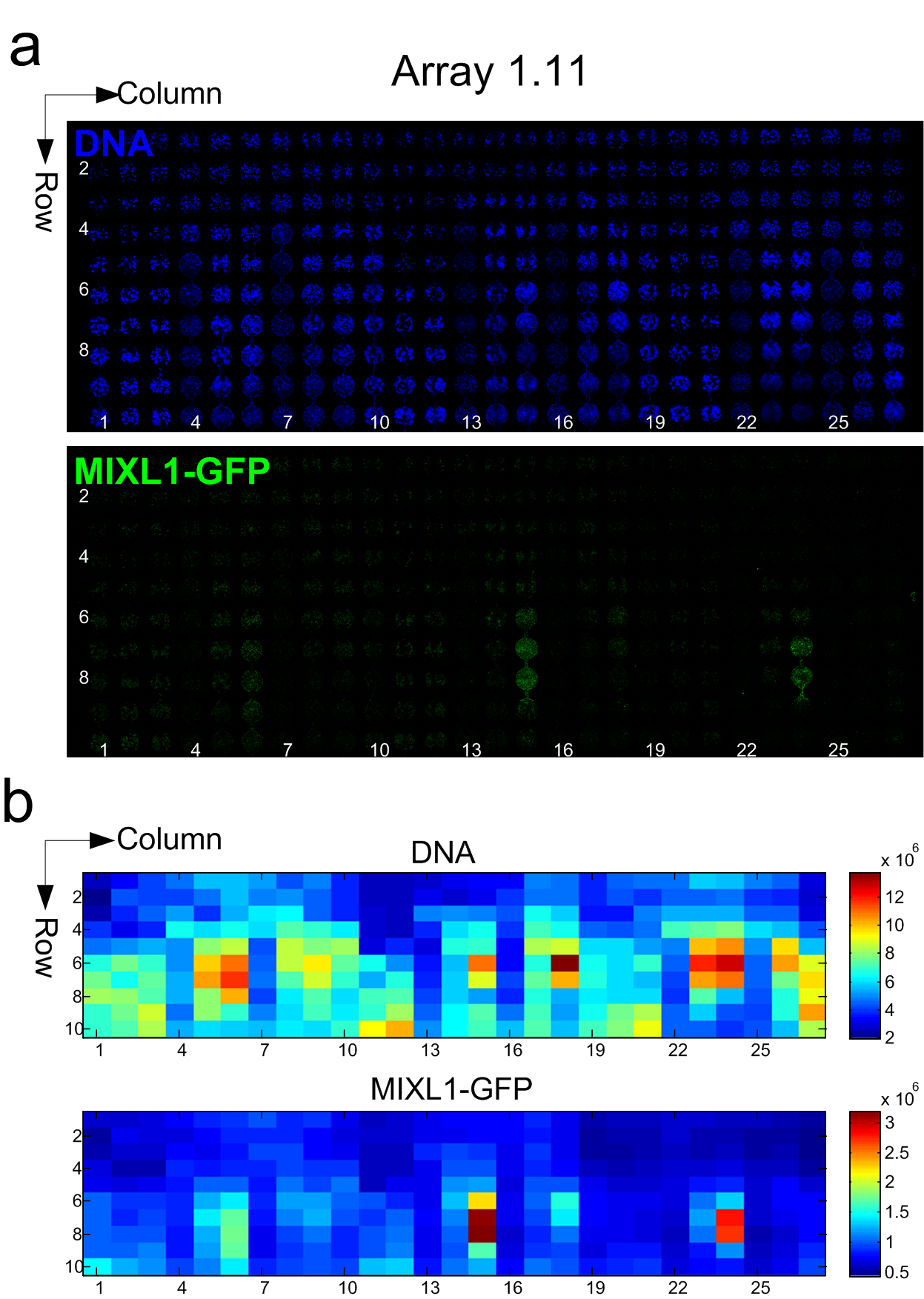

Supplement: Figure S5 — Array run for 3.5 d. (a) Confocal tile scan images of HES3(MIXL1GFP/w) hESCs expressing GFP and counterstained with Hoechst 33342 at 3.5 d experiment endpoint. Similar distributions of DNA and GFP intensities were observed as for arrays run for 2.5 d. Maximum intensity projection of a z-sectioned image is shown, and has been linearly enhanced for publication. Raw images were used for analysis. (b) Heatmaps of total fluorescence intensities in replicate arrays for each marker (arbitrary units). Similar distributions of DNA and GFP intensities were observed as for arrays run for 2.5 d. (TIF) [file pone.0052405.s005.tif]

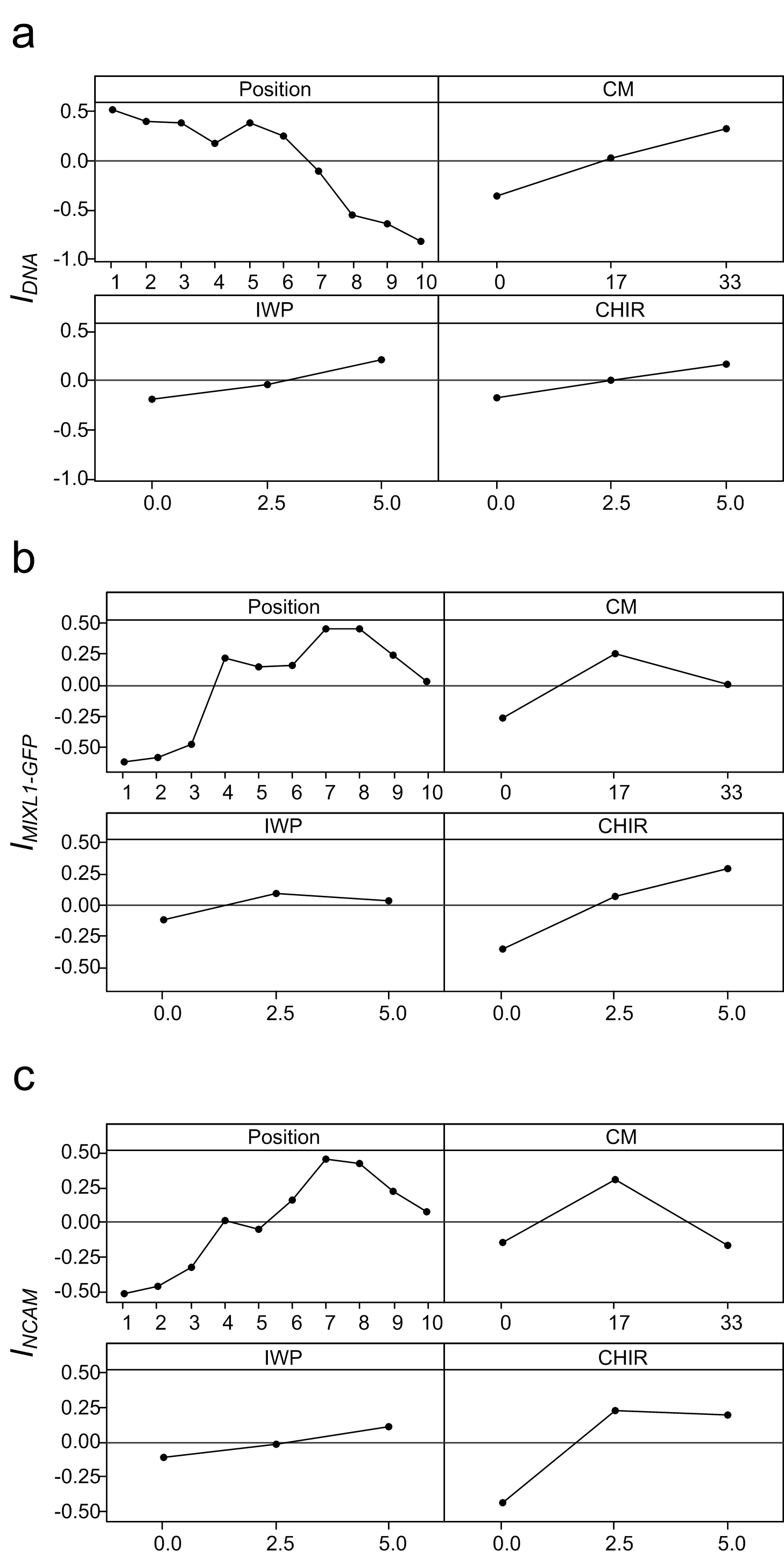

Supplement: Figure S6 — Factorial analysis of array data for HES3(MIXL1GFP/w) hESCs – main effects. (a-c) Main effects plots mapping effect magnitudes of the individual factors Position, CM, IWP and CHIR on IDNA (a), IMIXL1-GFP (b), and INCAM (c) expression indices. Units are ± global standard deviations relative to global mean for each marker. (TIF) [file pone.0052405.s006.tif]

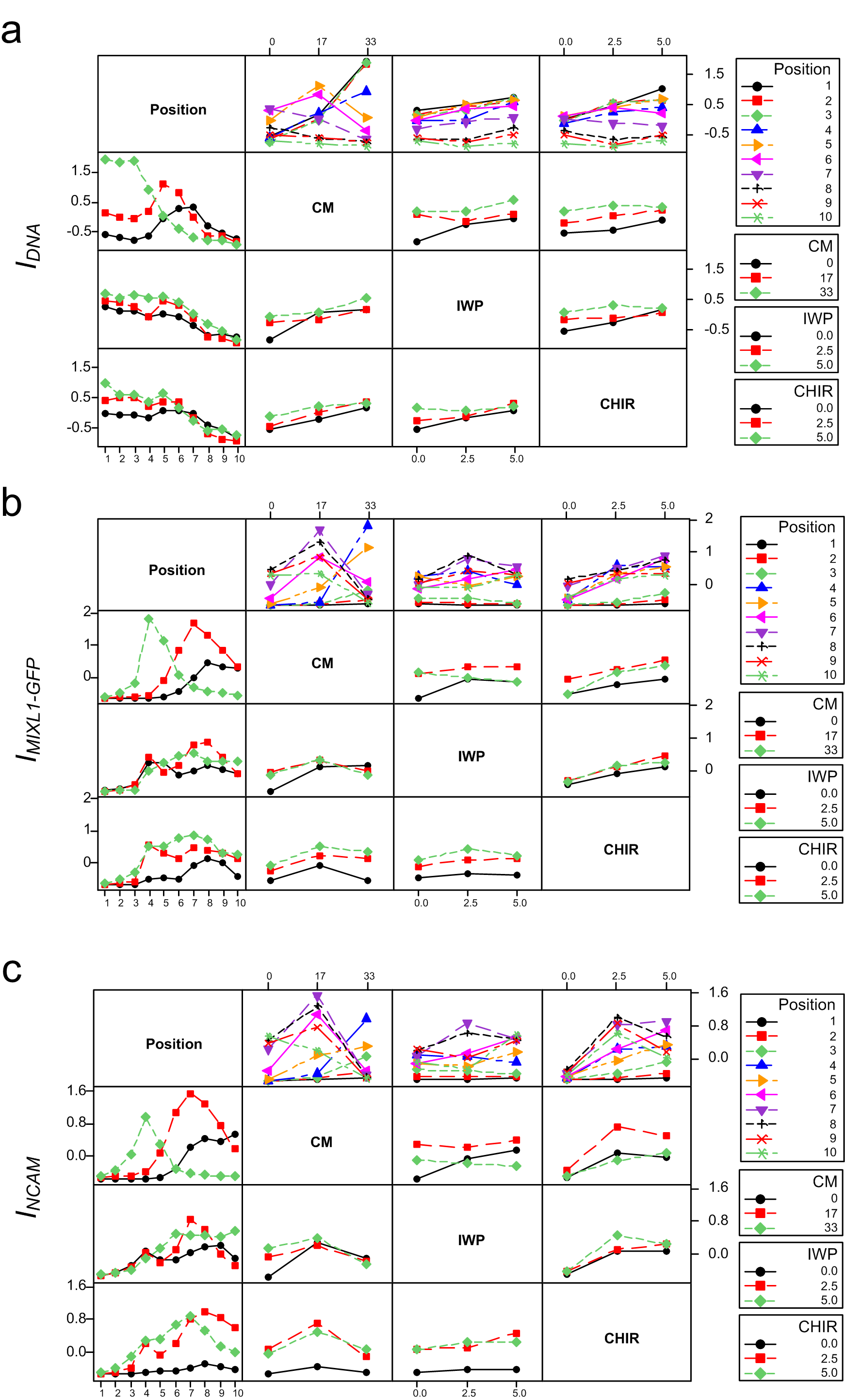

Supplement: Figure S7 — Factorial analysis of array data for HES3(MIXL1GFP/w) hESCs – interaction effects. (a-c) Interaction effects plots showing effect magnitudes for sets of two combined factors on expression index means for IDNA (a), IMIXL1-GFP (b), and INCAM (c) expression indices. Units are ± global standard deviations relative to global mean for each marker. (TIF) [file pone.0052405.s007.tif]

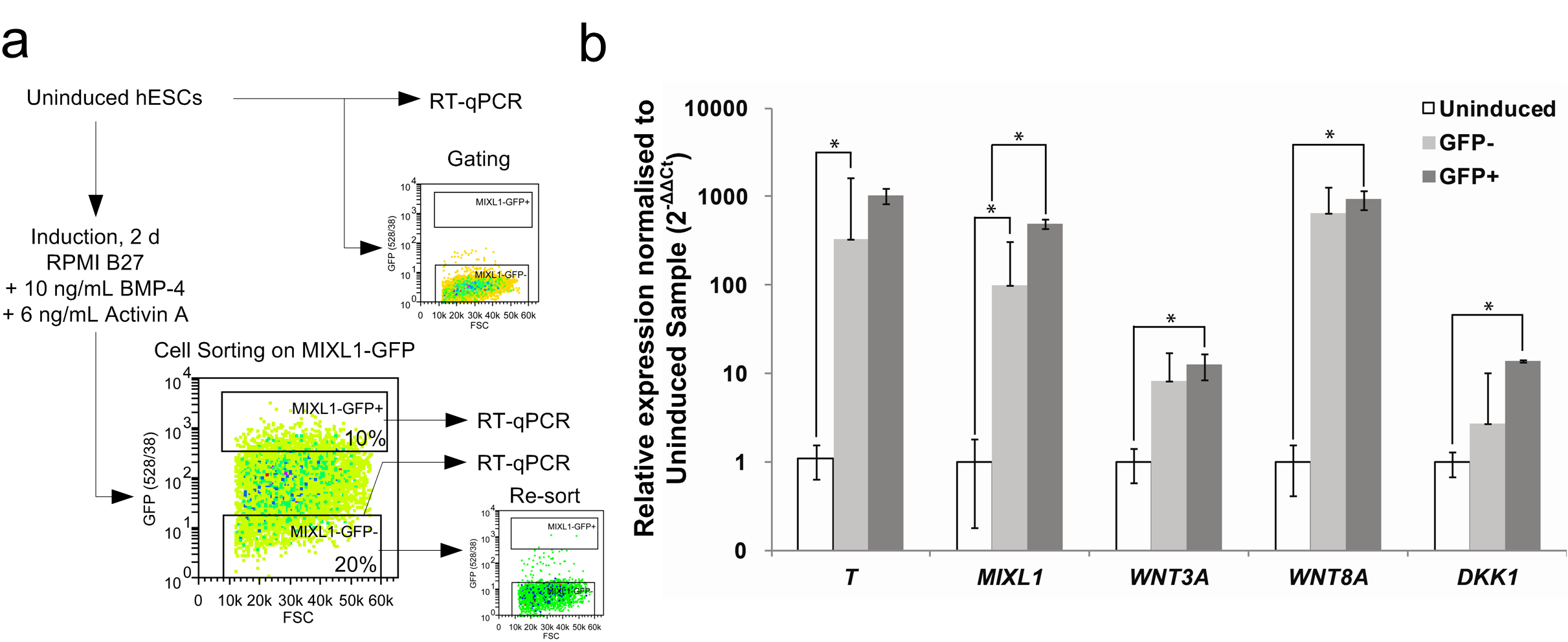

Supplement: Figure S8 — Gene expression profiling of MIXL1-GFP-sorted populations. (a) Cell sorting strategy. Live, single cells were identified using FSC and SSC area and width parameters and propidium iodide exclusion, and then the top 10% and bottom 20% of cells in terms of GFP-expression were sorted into separate fractions. (b) RT-qPCR quantification of gene expression in control (uninduced, unsorted) and 2 d induced, MIXL1-GFP-sorted cell populations. Bars represent mean ± s.e.m. (n = 6 from 3 independent sorts) of gene expression relative to GAPDH, normalised to undinduced samples. * indicates p<0.05, one-way ANOVA. Relative to uninduced cells, induced cells showed upregulation of mesendodermal markers T and MIXL1, soluble Wnt molecules WNT3A and WNT8A, and the soluble Wnt antagonist DKK1. (TIF) [file pone.0052405.s008.tif]
