# Supplementary material for: Microbioreactor Arrays for Full Factorial Screening of Exogenous and Paracrine Factors in Human Embryonic Stem Cell Differentiation
Source: PLoS One. 2012 Dec 26;7(12):e52405. doi: 10.1371/journal.pone.0052405 (PMC3530582; doi:10.1371/journal.pone.0052405)
Supplement: Table S1 — Physical Parameters. (DOC) [file pone.0052405.s009.doc]

| **Parameter** | **Unit** | **Microbioreactor** | **24-well Plate** |
| --- | --- | --- | --- |
|  |  |  |  |
| **Chamber Dimensions** |  |  |  |
| Diameter | mm | 1.63 | 15.49 |
| Culture Area | mm^2^ | 2.09 | 188.4 |
| Culture Area Specified | mm^2^ | - | 200 |
| Height | mm | 0.25 | 2.5 |
| Nominal Volume | μL | 0.52 | 500 |
| Surface-Area-to-Volume (SAV) Ratio | mm^2^/mm^3^ | 4 | 0.4 |
| Plate to Bioreactor Concentration Factor | - | 10 | 1 |
|  |  |  |  |
| **Column Dimensions** |  |  |  |
| Culture Area | mm^2^ | 21.4 |  |
| Height | mm | 0.25 |  |
| Volume | μL | 5.4 |  |
|  |  |  |  |
| **Array Dimensions** |  |  |  |
| Culture Area | mm^2^ | 579 |  |
| Height | mm | 0.25 |  |
| Volume | μL | 145 |  |
|  |  |  |  |
| **Nominal Flow Conditions** |  |  |  |
| Culture Area per Column | cm^2^ | 0.214 |  |
| Flowrate per Unit Area | μL/h/cm^2^ | 10 |  |
| Channel Flowrate | μL/h | 2.143 |  |
|  | m^3^/s | 5.95E-13 |  |
| Total Array Flowrate | μL/h | 57.851 |  |
| Individual Syringe Flowrate | μL/h | 9.642 |  |
|  |  |  |  |
| **^a^ Reynolds Number, Re** |  |  |  |
| Fluid Density, ρ | kg/m^3^ | 1000 |  |
| Average Velocity, *Q*/*A* (chamber at full width) | m/s | 1.461E-06 |  |
| Average Velocity, *Q*/*A* (interconnect) | m/s | 9.523E-06 |  |
| Hydraulic Diameter, *D_h_* (full width) | m | 4.34E-04 |  |
| Hydraulic Diameter, *D_h_* (interconnect) | m | 2.50E-04 |  |
| Fluid Viscosity, μ | Pa.s | 1.00E-03 |  |
| Average Re (chamber at full width) | - | 6.33E-04 |  |
| Average Re (interconnect) | - | 2.38E-03 |  |
|  |  |  |  |
| **^b^ Péclet Number, Pe** |  |  |  |
| Diffusivity (glucose) | m^2^/s | 6.00E-10 |  |
| Diffusivity (40kDa growth factor) | m^2^/s | 8E-11 |  |
| Average Pe (chamber at full width) (glucose) | - | 1.06E+00 |  |
| Average Pe (chamber at full width) (GF) | - | 7.91E+00 |  |
|  |  |  |  |
| **^c^ Shear Stress, τ** |  |  |  |
| Shear stress (chamber at full width) | Pa | 3.51E-05 |  |
| Shear stress (interconnect) | Pa | 2.29E-04 |  |

**Notes**

^a^ Calculated as , where density (ρ) = 1×10^3^ kg/m^3^; *Q* represents flowrate (m^3^/s); *D_h_* represents hydraulic diameter; viscosity (μ) = 10^-3^ Pa.s; and *A* represents cross-sectional area. Density and viscosity assumed as for water.

^b^ Calculated as , where *D_AB_* represents the diffusion coefficient.

^c^ Shear stress estimated as $\tau=6\mu Q/h^{2}w$, where height (*h*) *=* 2.5×10^-4^ m; and width (*w*) *=* 1.63×10^-3^ m for chamber at full width and 2.5×10^-4^ m for chamber interconnect.
