# Supplementary material for: Microbioreactor Arrays for Full Factorial Screening of Exogenous and Paracrine Factors in Human Embryonic Stem Cell Differentiation
Source: PLoS One. 2012 Dec 26;7(12):e52405. doi: 10.1371/journal.pone.0052405 (PMC3530582; doi:10.1371/journal.pone.0052405)
Supplement: Table S2 — RT-qPCR Primer Sequences. (DOC) [file pone.0052405.s010.doc]

| **Gene** | **Forward 5’-3’** | **Reverse 5’-3’** | **Product Size (bp)** | **Accession #** |
| --- | --- | --- | --- | --- |
| *T* | TCAGCAAAGTCAAGCTCACCA | CCCCAACTCTCACTATGTGGATT | 102 | NM_003181 |
| *MIXL1* | CCGAGTCCAGGATCCAGGTA | CTCTGACGCCGAGACTTGG | 58 | NM_031944 |
| *WNT3A* | AGCAGGCTCTGGGCAGCTACC | GCTGCGAGCCCAGGGAGGAA | 75 | NM_033131 |
| *WNT8A* | GAGCTGGGAACAGCGCCGAG | GCCCACAGCTACGTCGCTCC | 198 | NM_058244.2 |
| *DKK1* | GGAAGCGCCGAAAACGCTGC | TCTGGAATACCCATCCAAGGTGCT | 167 | NM_012242.2 |
